# Electronically Preresonant Stimulated Raman Scattering Microscopy of Weakly Fluorescing Chromophores

**DOI:** 10.1021/acs.jpcb.3c01407

**Published:** 2023-07-05

**Authors:** Andrea Pruccoli, Mustafa Kocademir, Martin J. Winterhalder, Andreas Zumbusch

**Affiliations:** Department of Chemistry, Universität Konstanz, 78464 Konstanz, Germany

## Abstract

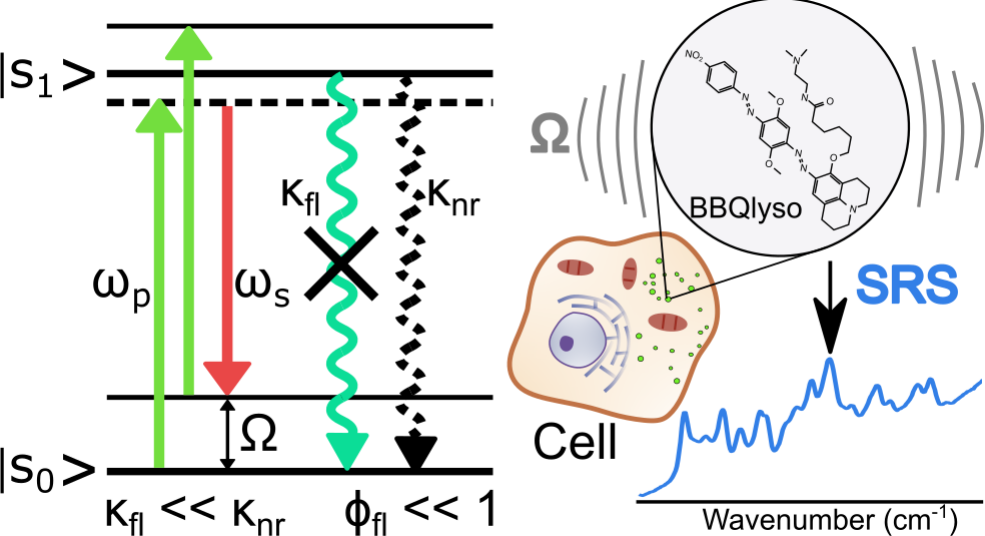

Stimulated Raman Scattering microscopy is an important imaging technique. Its broader application, however, is hampered by its comparatively low sensitivity. Using organic fluorophores, it has recently been demonstrated that, similar to spontaneous Raman microscopy, the sensitivity of stimulated Raman microscopy is increased by orders of magnitudes if electronic preresonances are exploited. In this Article, we show that this approach also works with low quantum yield chromophores. We investigate the relevant photophysics and discuss the background arising from preresonant excitation conditions. Applications of preresonant stimulated Raman scattering microscopy for the imaging of weakly fluorescing labels in fixed and live cells are demonstrated.

## Introduction

To date, confocal fluorescence microscopy arguably is the most important optical technique for the investigation of cell biological samples. It features high sensitivity down to the single molecule detection limit, high selectivity in combination with dedicated labeling techniques, and fast image acquisition times. Fluorescence-based microscopy, however, also has important drawbacks, such as the need for sample labeling, restricted capabilities for multicolor experiments due to the broad spectral width of normal fluorophores, and limited observation times caused by the photobleaching of fluorescent labels.

For specific applications, spontaneous Raman microscopy can therefore be an interesting complement to fluorescence imaging. In Raman microscopy, contrast is generated on basis of the Raman scattering intensity of molecular vibrations. Since even small molecules possess vibrational spectra with many narrow bands, vibrational spectroscopy is well suited for the differentiation between various sample molecules. Additionally, typically no labeling is necessary, and photobleaching is of no concern. However, Raman microscopy has limitations preventing its broader application. Due to the low Raman scattering cross-sections, its sensitivity is approximately 6 orders of magnitude lower than that of fluorescence microscopy, image acquisition times are long, and Raman detection in complex environments such as biological cells is often hampered by sample background fluorescence. Therefore, a number of methods have been developed to push the limits of Raman based imaging techniques. Image acquisition times can be significantly reduced by using techniques such as slit scanning^1,2^ or compressive Raman,^3,4^ and surface or electronic resonance enhancement often in combination with Raman labels is used to improve selectivity^5,6^ and sensitivity.^7^

In addition to these methods, nonlinear Raman microscopy has become an important technique for the investigation of biological and material scientific samples.^8−11^ Initially, coherent anti-Stokes Raman scattering (CARS) microscopy^12,13^ was introduced as a means for the fast imaging of unlabeled samples. Since CARS microscopy suffers from a nonresonant background signal and a quadratic dependence on sample molecule concentration, soon afterward, stimulated Raman scattering (SRS) microscopy was introduced.^14−16^ SRS is free of a purely electronic background and has a linear dependence on the scatterer concentration. Both CARS and SRS microscopy allow faster image acquisition than spontaneous Raman microscopy, because in most implementations only one vibrational frequency is selectively monitored at a time. The sensitivity of both methods, however, is similar to that of spontaneous Raman microscopy^17^ such that, in general, concentrations larger than 1 mM are needed at an imaged voxel to generate a nonlinear Raman signal above the noise level.

As is the case for spontaneous Raman scattering, nonlinear Raman scattering processes can be enhanced significantly if electronic resonances of the sample molecules are exploited. In spontaneous Raman scattering, this is observed when the excitation wavelength approaches an electronic transition of the sample.^18^ The situation is somewhat more complicated in CARS and SRS microscopy, since for both methods, two laser beams are used for the excitation. This gives rise to several resonance terms involving electronic states.^19^ Obviously, signal enhancement due to electronic resonances requires the presence of a chromophore with a suitable π system. Unless the sample naturally contains chromophores, this means that spontaneous or nonlinear Raman microscopy with electronic enhancement requires the use of labels. Then, however, vibrational Raman signal intensities comparable to those of fluorescence signals can be obtained.

The use of Raman probes with electronic preresonances (EPR) has recently been demonstrated for spontaneous Raman^7^ and for SRS microscopy.^20,21^ In the first case, strictly low quantum yield molecules had to be used to allow detection of the Raman signals against the sample molecules’ fluorescence background. By contrast, background fluorescence is of no concern in SRS microscopy, and the vast majority of EPR-SRS microscopy experiments reported to date have been limited to the use of fluorescent labels. Here, we report EPR-SRS microscopy with weakly fluorescing labels. Their use promises to significantly extend the range of accessible molecules, especially to low quantum yield chromophores that occur naturally in biological samples. Time-resolved SRS spectroscopy allows us to get insight into different processes contributing to the SRS signal and the background. As the potentially higher resistance of low quantum yield molecules to photobleaching would be of great interest, we also investigated the bleaching behavior of four commercially available low quantum yield marker molecules. Finally, we demonstrate how these molecules can be employed to selectively image different cellular structures in fixed and live cells.

## Experimental Section

All chemicals were purchased from commercial sources and used without any further purification. Stock solutions of the quencher chromophores were prepared by dissolving the compounds in deuterated DMSO (DMSO-*d*_6_), and the concentration were measured by UV–vis absorption after appropriate dilution.

### Cell Culture

All cells were cultured in Dulbecco’s modified Eagle’s medium (DMEM, Thermo Fisher Scientific), 10% v/v fetal calf serum, and 1% penicillin/streptomycin (Thermo Fisher Scientific). During incubation, the temperature was kept at 37 °C, and the atmosphere contained 5% CO_2_. Each microscope sample was prepared in an imaging dish with a 35-mm-diameter glass bottom (μ-dishes 35 mm, ibidi) coated with poly-l-lysine (molecular weight 70 000–150 000, Sigma Aldrich). For fixation, the cells were washed with PBS and fixed using 4% paraformaldehyde (PFA) in PBS for 15 min at room temperature.

### Lysosomal Staining

The live cell lysosomal tracker BBQlyso was synthesized by modifying the BBQ chromophore (cf. below) with a diamine following the procedure described by Kuzmin et al.^7^ One day before staining, 100 000 cells were transferred to the μ-dishes (ibidi) and incubated at 37 °C and 5% CO_2_. The cells were then stained with BBQlyso to reach a concentration of approximately 0.7 μM for 15 min. Subsequently, the samples were washed with phosphate-buffered saline solution (PBS) and imaged directly afterward in 2 mL of DMEM. For staining with LysoTracker Green (Thermo Fisher Scientific) and LysoTracker Deep Red (Thermo Fisher Scientific), the same procedure as described for BBQlyso was applied; however, in theses cases, the cells were exposed to a dye concentration of 0.5 μM for 15min.

### Endoplasmic Reticulum Staining

The cells were fixed with 1 mL of 4% PFA for 15 min before permeabilization with 1 mL of 0.5% Triton-x in PBS for 10 min at room temperature. The cells were then stained with 1 μM of QSY21 for 20 min. Finally, the cells were washed three times with PBS and imaged in 2 mL of PBS.

### Nuclear Staining

The cells were incubated for 24 h with 50 μM 5-azidomethyl-2′-deoxyuridine (AmdU) in 2 mL of DMEM medium. After incubation, the samples were washed three times with PBS, and the procedure for fixation and permeabilization was carried out as described above. The cells were then incubated with 30 μM of a solution of the conjugate of the dye and dibenzo-bicyclo-octyne (DBCO) for 2 h. After staining, the samples were washed three times with PBS before imaging in 2 mL of PBS.

### Cell Staining with RedDot2

The cells were fixed with 1 mL of 4% PFA for 15 min before permeabilization with 1 mL of 0.5% Triton-x in PBS for 10 min at room temperature. The cells were then stained with a 1:200 diluted standard solution of RedDot2 (Biotium, Inc., US) for 15 min. Finally, the cells were washed three times with PBS and imaged in 2 mL of PBS.

### Stimulated Raman Scattering and Fluorescence Measurements

SRS experiments were performed using two different setups. Time-resolved SRS spectroscopy was done using a home-built Er:fiber laser-based SRS microscope that was described in detail earlier.^22^ In this case, a pump laser with a repetition rate of 39.32 MHz with its wavelength fixed at 771 nm and a Stokes beam tunable between 828 and 890 nm and modulated at 19.66 MHz were used. The pump power at the sample is 3.2 mW, while the Stokes power is variable between 3 and 8 mW. The Stokes power is recorded by a photodiode and can be used to normalize the SRL spectrum. The mentioned wavelengths correspond to a vibrational spectral range between 900 and 1730 cm^–1^. Durations for pump and Stokes pulses were 3 ps. All SRL spectra from this setup are recorded with a 20 ms time constant.

SRS microscopy was done using a commercial confocal laser scanning microscope with SRS capability (TCS SP8, Leica Microsystems). The system works with a fixed Stokes beam modulated at 20 MHz and with a wavelength of 1031 nm and a pump beam modulated at 80 MHz and tunable between 720 and 980 nm. This allows the recording of SRL spectra in the vibrational spectral region between 500 and 4200 cm^–1^. The maximum power is set for the Stokes beam at 300 mW and 150 mW for the pump beam. Depending on the measurement, this power was usually attenuated to less than 50% of the maximum to avoid thermal damage. The beams were spatially and temporally overlapped and focused on the sample using a water immersion objective (25×, NA 0.95, HCX IRAPO L, Leica Microsystems). After passing through the sample, the signal is collected with an oil condenser (NA 1.4, Leica Microsystems). The pixel dwell time is kept at 20 us/pixel to maintain a compromise between speed and noise, and the time constant was 10 μs. The data were processed using commercial software (LAS X, Leica Microsystems). The SP8 microscope was also employed for fluorescence imaging using four different solid state laser lines at 488 nm, 514 nm, 552 nm, and 638 nm. Fluorescence was detected by a hybrid detector (HyD, Leica Microsystems).

## Results and Discussion

**Figure 1:**
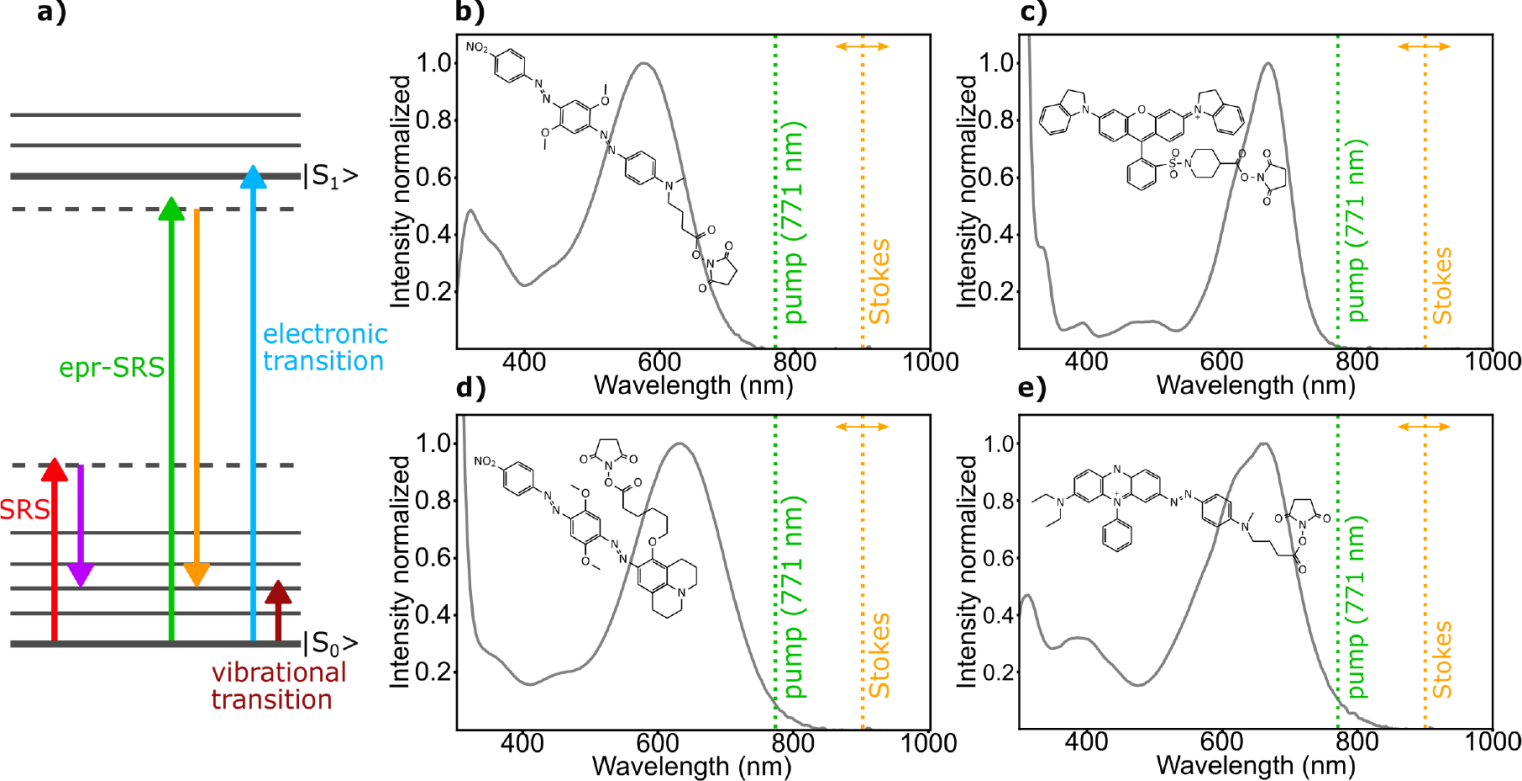
(a) Energy diagram for EPR-SRS. (b–e) Chemical structures of the studied quenchers in the N-hydroxysuccinimide form and the respective absorption spectra. (b) Black Hole 2 (BH2), (c) QSY21, (d) Black Berry Quencher (BBQ), (e) Black Hole 3 (BH3)

In SRS, a pump and a Stokes laser are used to excite a sample (Figure 1a). If the frequency difference between the two lasers coincides with the energy of a vibrational transition of the sample, stimulated Raman scattering occurs. It can be detected either as a loss in the number of pump photons (stimulated Raman loss, SRL) or as a gain in the number of Stokes photons (stimulated Raman gain, SRG). For the intensities of the SRL and SRG signals, one finds *I*_SRL_ ∝ −*I*_p_*I*_s_*N*σ and *I*_SRG_ ∝ *I*_p_*I*_s_*N*σ.^19^ Here, *I*_p_ and *I*_s_ are the respective intensities of the exciting pump and Stokes lasers, *N* is the number density of scatterers, and σ is the Raman scattering cross-section. In most SRS experiments, the excitation frequencies ω_p_ and ω_s_ are chosen far from electronic transition frequencies ω_0_ of the sample molecules. By contrast, EPR-SRS exploits the fact that the Raman cross-section σ is frequency-dependent, increasing with (ω_0_ – ω_p_)^−4^. Since ω_p_ is usually closer to the electronic transition frequency than ω_s_, its respective resonance enhancement term is stronger than that of ω_s_, which we neglect here. In practice, the SRS detection sensitivities grow by 3 to 4 orders of magnitude if the pump laser frequency ω_p_ is chosen close to the electronic transition energy ω_0_.^21,23^ The choice of ω_p_, however, is also influenced by the necessity to keep the direct electronic excitation low since electronic absorption inevitably leads to photobleaching of the sample molecules. Thus, a compromise between high electronic preresonance enhancement of the Raman scattering cross-section and the minimization of electronic absorption has to be found. Despite these efforts, however, some degree of photobleaching will be observed in all EPR-SRS experiments. Electronic absorption lines are typically described by a Voigt profile, i.e., the convolution of a Lorentzian line shape originating from the homogeneous line broadening and a Gaussian line shape reflecting the static inhomogeneous broadening contributions. Wei and Min^21^ claim that, for the dyes they investigated, the Voigt profile decays faster than the Raman enhancement and that choosing a pump frequency at roughly ω_0_ – ω_p_ = 3 Γ, where Γ is the full width at half-maximum of the electronic absorption band, provides a good compromise between absorption and enhancement. This would require that inhomogeneous broadening with its exponentially decaying line shape dominates. In general, electronic absorption bands are composed of several vibronic bands with their intensities scaled by the respective Franck–Condon factors. In room temperature solutions of organic chromophores, the homogeneous line width of each vibronic transition is typically comparable to the inhomogeneous broadening.^24−26^ Thus, electronic transitions of room temperature chromophore solutions are commonly well described by the Lorentzian shapes of homogeneous lines. Since these have a similar frequency dependence as the preresonance enhancement, there is no generally valid expression for finding the best compromise between avoidance of absorption and maximizing Raman enhancement. Instead, this sweet spot has to be found for each chromophore individually with its resistance against photobleaching being the most important aspect. It should be emphasized here that the discussion is restricted to the low energy side of the purely electronic 0–0 transition since all other vibronic transition from the vibrational ground state of the |*S*_0_⟩ state will have higher energies such that their enhancements will be low. Vibronic transitions from thermally excited vibrational states of |*S*_0_⟩, by contrast, can be neglected because their Boltzmann population is too low.

Since there is no connection between a molecule’s fluorescence quantum yield and the electronic preresonant enhancement of its Raman scattering, enhancement is observed for fluorescent and weakly fluorescing molecules alike. With the exception of a recent work on the SRS detection of retinoids,^27^ however, low quantum yield chromophores have not yet been investigated with EPR-SRS microscopy. For our work, we selected four low quantum yield molecules absorbing in the red spectral range that are commercially available as fluorescence quenchers (Figure 1b–e). We use these chromophores to investigate the photophysical behavior relevant for the imaging of weakly fluorescing chromophores with EPR-SRS microscopy and to demonstrate their usefulness as Raman labels in experiments with fixed and live biological cells. The compounds are Black Berry Quencher (BBQ, absorption maximum at 625 nm), Black Hole 2 (BH2, absorption maximum at 580 nm), Black Hole 3 (BH3, absorption maximum at 660 nm), and QSY21 (absorption maximum at 670 nm). With the exception of QSY21, all of these dark quenchers are azo chromophores, whereas QSY21 is a rhodamine chromophore substituted at the amine positions with two indoline groups. The spontaneous Raman spectra of all quencher molecules are collected in Figure S1.

In the azo compounds, fluorescence quantum yields most likely are very low due to the efficient relaxation via cis–trans isomerization at the azo double bond. For this class of compounds, isomerization time constants are known to lie in the picosecond range.^28^ By contrast, comparison with other rhodamines shows that, in the case of QSY21, efficient relaxation from the first excited electronic state is expected to be mediated by the rotation of the indoline substituents.^29^ From the absorption spectra, a moderate preresonance enhancement is expected for BH2, whereas the other quenchers investigated should exhibit a stronger enhancement due to their longer wavelength absorption.

### Time-Resolved SRS Spectroscopy

**Figure 2:**
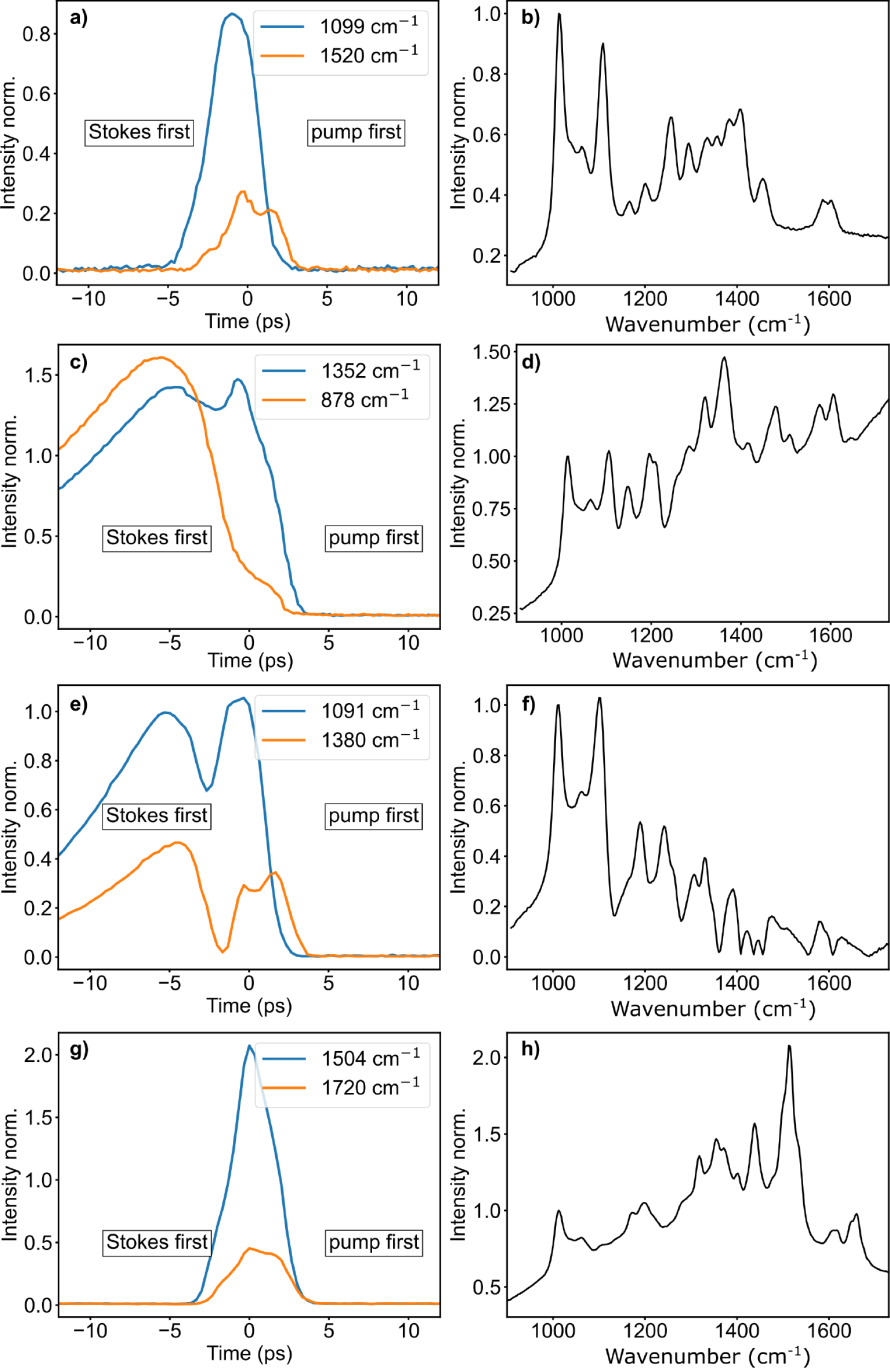
Time-resolved EPR-SRL detected signals (left) and EPR-SRL spectra (right) recorded with a pump wavelength at 771 nm and varying Stokes wavelengths of 1 mM solutions of different quenchers. Blue lines in the time-resolved data were recorded at vibrational resonances; orange lines were recorded off-resonance. BH2 (a,b), BH3 (c,d), BBQ (e,f), QSY21 (g,f). All data are normalized to the CD_3_ peak of the solvent DMSO-*d*_6_ at 1007 cm^–1^.

**Figure 3:**
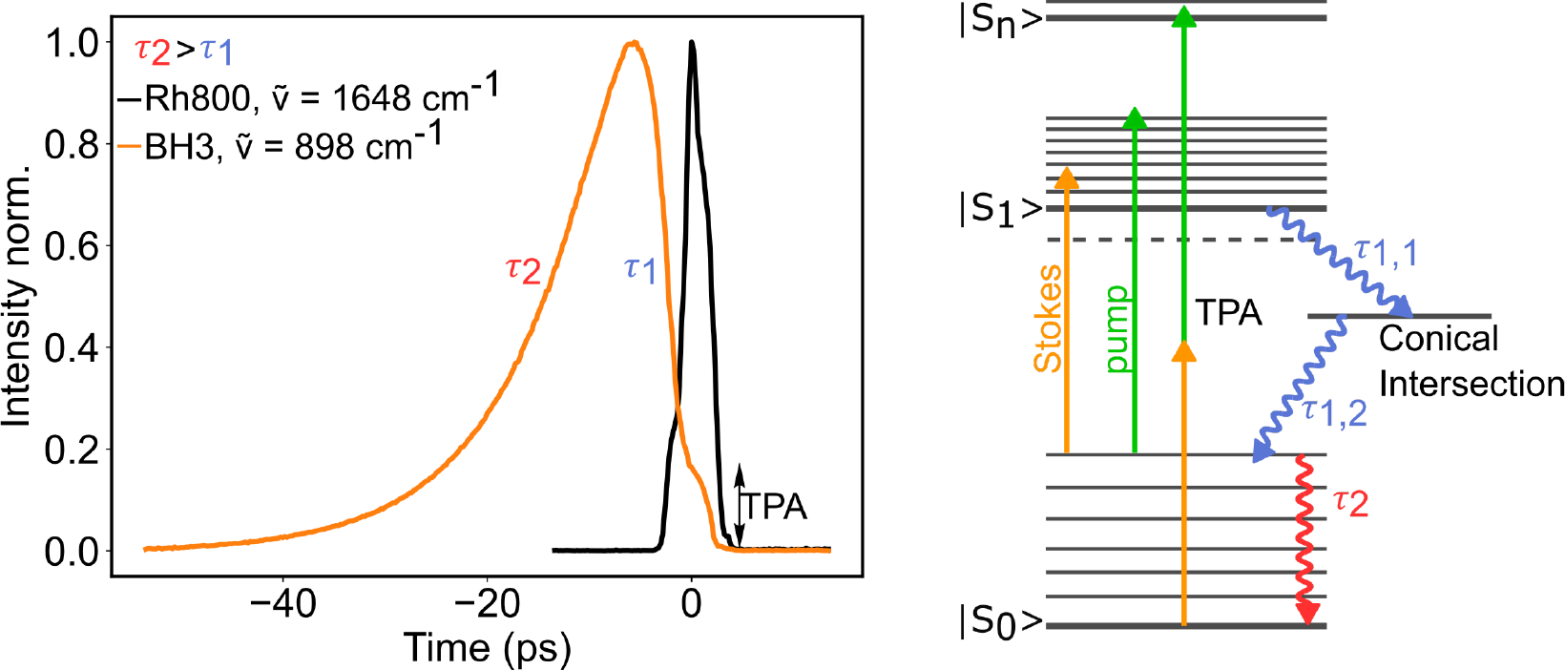
Time-resolved SRS spectra of Rh800 (black) and BH3 (orange) normalized at maximum intensity and energy diagram characterization of BH3 background processes, two-photon absorption (TPA), and Stokes-dependent pump absorption.

We first recorded time-resolved SRS spectra of solutions of the various chromophores (left column in Figure 2). Variation of the delay between the picosecond pump (wavelength λ_p_ = 771 nm) and Stokes (wavelength λ_s_ = 830–890 nm) pulses gives insight into the nature of the background signals against which SRS is detected. It is important to note that all data are acquired using lock-in detection with the Stokes beam modulated at half the repetition rate of the pump beam. Therefore signals are only detected if they originate from the interaction of pump and Stokes photons. A background signal that is always present at time *t* = 0 is due to mixed two-photon absorption (TPA), i.e., simultaneous absorption of a pump and a Stokes photon (Figure 2, left column). The loss of a pump photon in this process appears as a background signal in SRL detection. Since in TPA no intermediate state with a lifetime is present, the respective signal has a temporal response equivalent to a cross-correlation of the two exciting laser pulses. This signal with the same temporal behavior is seen for all chromophores tested. For BH3 and BBQ, an additional background signal is observed at negative times, meaning that the Stokes pulse arrives before the pump pulse. Based on previous studies on azo-benzene,^28^ we postulate that this signal is due to the initial absorption of a Stokes photon from the red tail absorption of the chromophore. The red absorption and excitation into the |*S*_1_⟩ state (Figure 3) can be due either to the molecules residing in higher vibrational states or to subpopulations of molecules with higher conformational energy in the red tail of the absorption peak. This process involves one Stokes photon as confirmed by the linear dependence of the compounds’ weak fluorescence intensities on the Stokes excitation power (Figure S2). Subsequently, the molecule can undergo fast relaxation to the conical intersection and then to the electronic ground state |*S*_0_⟩. This process appears to have lifetimes too short to be resolved by our instrument, τ_1,1_ + τ_1,2_ < 3 ps. After relaxation to the |*S*_0_⟩ state, the molecule is still in higher vibrational states from where it can efficiently absorb a pump photon, thus producing the observed background signal at negative times in SRL detection (Figure 3). The same process will take place at positive times starting with a pump photon. Since this will occur at the pump frequency, it will not show up in SRL detection due to the lock-in filtering at the Stokes modulation frequency. The time-dependent background is shown to be linearly proportional to the pump photon intensity (Figure S2). Tail-fitting of the decay to larger negative times yields time constants (τ_2_) of 9.0 ps for BH3 and 5.7 ps for BBQ (Figure S3). This is in accordance with reported time constants for the thermal cooling of azo-benzenes.^28,30^

The suitability of the various chromophores for highly sensitive detection in imaging applications can be estimated by comparing the signals recorded at time *t* = 0 for vibrational resonance and off-resonance (blue and orange lines, respectively in Figure 2). We find that despite the strong background present for BH3, the highest contrasts at similar concentrations are obtained for BH3 and QSY21.

### SRS Spectra in the Fingerprint Region

We next recorded SRL spectra for all chromophores dissolved in DMSO-*d*_6_ by setting the temporal delay to *t* = 0 and tuning the Stokes laser wavelength from λ_s_ = 828 to 887 nm. This corresponds to Raman shifts ranging from 900 to 1700 cm^–1^. As discussed in the paragraph on the time-resolved data, all chromophores are affected by a wavelength dependent background mostly due to mixed TPA. Depending on the electronic TPA absorption spectrum, this background is either slowly rising or declining with increasing Raman shift in the SRL spectra. For all chromophores, vibrational features are easily detected in their fingerprint SRL spectra. Note that the peak at 1007 cm^–1^ corresponds to the CD_3_ peak of the solvent DMSO-*d*_6_.^31^ All azo compounds feature an intense band at 1100 cm^–1^. As a comparison, the resonance Raman spectrum of *trans*-azobenzene^32^ features an intense peak at 1143 cm^–1^ that is due to a combination of bending and stretching vibrations. A further analysis of the numerous weaker bands present in the spectra of the three azo compounds goes beyond the scope of this work. The spectra reveal that a somewhat higher contrast and therefore also sensitivity in imaging applications can be expected for BH3 and BBQ compared to that for BH2. The rhodamine chromophore QSY21 shows the most intense SRL signal among all of the studied quenchers, especially when we consider its peak at 1510 cm^–1^ that is the most prominent peak at preresonance excitation wavelengths. Spontaneous Raman spectra show that this peak’s relative intensity decreases if the excitation wavelength is shifted to higher energies than the absorption maximum (cf. spontaneous Raman data for 488 and 785 nm excitation in Figure S1). This behavior has previously been described for Rhodamine 6G.^33^

### Photobleaching Behavior

The use of electronic preresonance enhancement strongly boosts the sensitivity of SRS microscopy, but working under preresonance Raman enhancement conditions inevitably also leads to electronic excitation of the sample molecules via one- or multiphoton absorption. The chromophore can then release the excitation energy either by internal conversion or by fluorescence. While fluorescence emission is no problem for SRS detection, potential photobleaching after electronic excitation destroys the sample molecules. Therefore, the stability of the sample molecules against photobleaching is an important aspect in EPR-SRS microscopy. Despite the fact that the first publications on EPR-SRS microscopy reported negligible photobleaching,^20^ in our experience EPR-SRS microscopy is always accompanied by significant photobleaching. The investigation of photobleaching mechanisms has been an important topic in confocal and two-photon fluorescence microscopy as well as in super-resolution microscopy.^34^ Most of the known photobleaching mechanisms proceed via an intermediate population of the |*S*_1_⟩ state. It is thus tempting to assume that short dwell times of the sample molecules in the |*S*_1_⟩ state lead to high photostability. The fact that the chromophores investigated in this work all have negligible fluorescence quantum yields means that they all possess high nonradiative decay rates and consequently low lifetimes of the |*S*_1_⟩ state. Measurements of the fluorescence lifetimes of the quencher molecules yields |*S*_1_⟩ lifetimes of 208 ps (BH2), 28 ps (BH3), 138 ps (BBQ), and 105 ps (QSY; Figure S4). To find out whether these short lifetimes indeed lead to the postulated higher photostability, we performed cw bleaching experiments of solutions of the quencher molecules and of four other well-known chromophores with comparable absorption wavelengths (Figures S5, S6). As postulated, we find that the photostability of the quencher molecules under cw excitation conditions is significantly larger than that of the fluorophores. Nevertheless, we observed strong decreases of the SRL signal intensity in EPR-SRS microscopy of all chromophores investigated (data not shown). This means that, despite the fact that the excitation pulses used in our EPR-SRS experiments were comparatively long for multiphoton imaging experiments (3 ps), nonlinear processes appear to be dominating the photobleaching of the quenchers under EPR-SRS imaging conditions. A similar behavior has been reported previously for fluorescein, indo-1, and aminocoumarin.^35^

### EPR-SRS Imaging of Fixed and Live Cells

From the discussion of their photophysical properties, it is evident that low quantum yield chromophores are good candidates for imaging applications using EPR-SRS. Therefore, we investigated the rhodamine chromophore QSY21 and the azo chromophore BBQ in live and fixed cell imaging experiments. These experiments were complemented by similar experiments with the intercalating chromophore RedDot2 that exhibits a strong increase in fluorescence quantum yields upon intercalation and with MB 660R as a standard commercial red fluorescent chromophore. The imaging experiments were carried out with the Leica TCS SP8 microscope. The longer pump wavelengths of this microscope result in a decrease of resonance enhancement. BBQ, for example, shows an SRL signal loss of around 30% (Figure S7).

**Figure 4:**
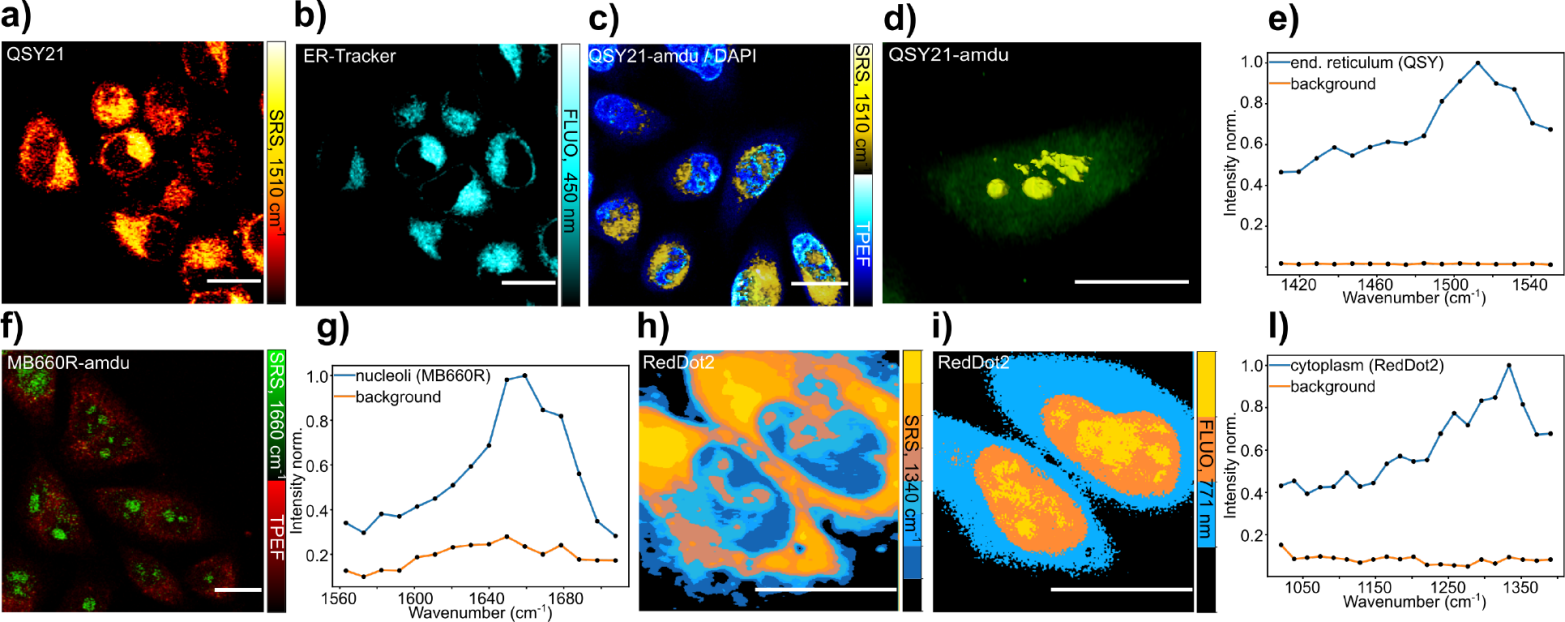
(a,b) EPR-SRS and two photon absorption fluorescence imaging of endoplasmic reticulum in fixed HeLa cells costained with QSY21 and ER tracker and (c) that of fixed HeLa cells costained with QSY21-AmdU (cytoplasm and nucleoli) and DAPI (nucleus). (d) 3D rendering of the EPR-SRS image obtained of a fixed HeLa cell stained with QSY21-AmdU. (e) EPR-SRS spectrum of HeLa cells stained with QSY21. (f) EPR-SRS and cellular autofluorescence imaging of fixed HeLa cells labeled with MB660R-AmdU. (g) EPR-SRS spectrum of MB660R-AmdU labeled HeLa cells. (h,i) Intensity segmented image of fixed HeLa cell stained with RedDot2 and imaged simultaneously with EPR-SRS and fluorescence. (i) EPR-SRS spectrum of HeLa cells stained with RedDot2. Scale bars: 20 μm.

First, we used QSY21 to stain fixed cells. EPR-SRL images were recorded by tuning the energy difference between the pump and Stokes beam to 1510 cm^–1^, which is the vibrational resonance detected in the solution EPR-SRL spectra (cf. Figure 2h). The corresponding peak is also detected in SRL spectra of the QSY21 labeled cell (Figure 4e). Costaining of the cells with the commercial endoplasmic reticulum (ER) tracker, ER-T blue-white, indicates that QSY21 localizes to the ER (Figure 4a,b).

To demonstrate the suitability of the low quantum yield molecules investigated here for use in combination with bioorthogonal labeling approaches, we next used a strain-promoted alkyne azide cycloaddition to click QSY21-DBCO to AmdU-treated fixed HeLa cells. AmdU is incorporated into nascent DNA, but due to the steric hindrance of the DBCO group, the reaction will mostly occur on single stranded DNA with the highest concentration in the nucleoli.^36^ EPR-SRS imaging of the cells costained with DAPI was performed at a Raman shift of 1510 cm^–1^. It shows that QSY21-DBCO is present in the whole nucleus but has a stronger localization to nucleoli than DAPI. As a comparison, we performed similar experiments with the commercial fluorescent dye conjugate MB 660R-DBCO that was also clicked to AmdU-treated fixed HeLa cells. The EPR-SRL spectrum shows an intense peak at 1660 cm^–1^ that was chosen for imaging (Figure S8). The cellular autofluorescence image from two-photon excited fluorescence (TPEF) using the pump laser was acquired simultaneously. MB 660R-DBCO appears to be even more localized to the nucleoli than QSY21-DBCO but otherwise gives similar results.

Especially for nuclear staining, intercalating dyes find widespread use. Chromophores of this type are of interest in this study of EPR-SRS detection of low quantum yield chromophores, since upon binding to DNA, the fluorescent quantum yields of intercalating dyes typically change drastically such that compounds which are hardly fluorescent in solution become good fluorophores upon binding. Since the EPR-SRS signal of intercalating dyes does not depend on the binding status, a comparison of EPR-SRS and fluorescence images of such compounds can give interesting insight into the distribution of the intercalator in cells. As an example, we studied the far red commercial nuclear stain RedDot2. The EPR-SRL spectrum of RedDot2 exhibits a peak at 1340 cm^–1^ (Figure S8). The comparison of the EPR-SRL imaging at this Raman shift with the two-photon excited fluorescence signal indeed clearly shows that RedDot2 is mainly localized in the cytoplasm and in the nucleoli where strong EPR-SRL signals are detected. The strong fluorescence signal of RedDot2 in the nucleus, by contrast, can mainly be attributed to intercalation.

**Figure 5:**
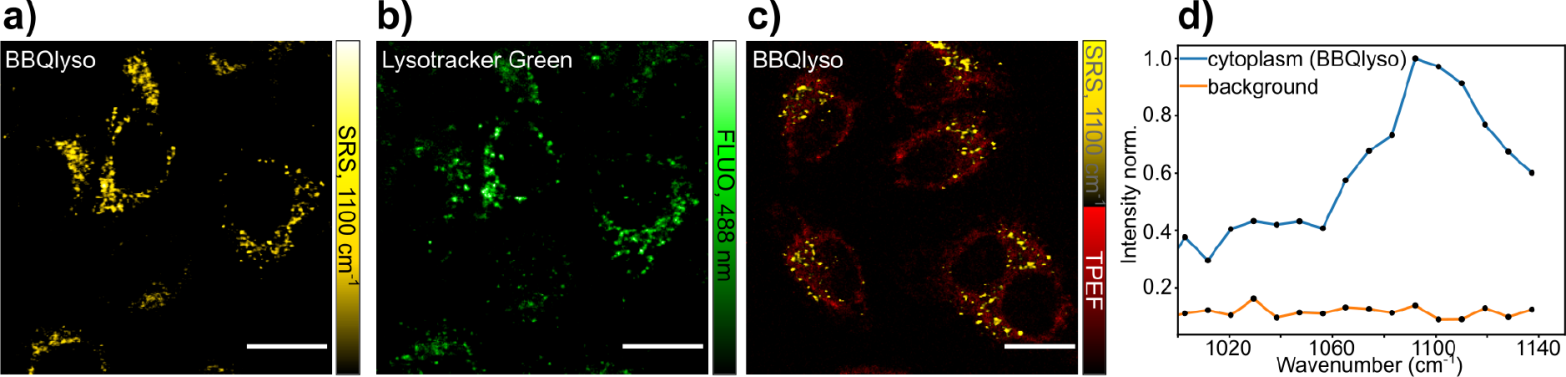
(a,b) EPR-SRS and fluorescence imaging of live HeLa cells costained with BBQlyso and Lysotracker Green. (c) EPR-SRS and autofluorescence imaging of live HeLa cells stained with BBQlyso. Scale bars: 20 μm. (d) EPR-SRS spectrum of BBQlyso labeled live HeLa cells.

We finally explored the use of low quantum yield chromophores in live cell EPR-SRS microscopy experiments. Following the procedure described by Kuzmin et al.,^7^ we modified BBQ with a diamine to obtain Black Berry LysoTracker (BBQlyso), a compound that accumulates in lysosomes. This modification of BBQ does not affect the EPR-SRL spectrum such that BBQlyso has the peak at 1100 cm^–1^ also present in the BBQ spectrum. BBQlyso was used to label live HeLa cells that were costained with the fluorescent commercial lysosomal stain, Lysotracker Green. The comparison of the EPR-SRL image recorded at a vibrational resonance of 1100 cm^–1^ with the fluorescence image of Lysotracker Green excited at 488 nm is shown in Figure 5. Clearly both signals colocalize, thus demonstrating the usefulness of BBQlyso as a live cell lysosomal marker for EPR-SRL imaging.

## Conclusion

In conclusion, we present investigations of the imaging of low quantum yield chromophores with EPR-SRS microscopy. Time-resolved SRL measurements show that the exploitation of preresonance electronic Raman enhancement in EPR-SRS is accompanied by two different types of background not present in normal SRS microscopy. It is present for both fluorescing and weakly fluorescing chromophores, and its strength cannot be predicted but must be investigated separately for every type of chromophore. Unfortunately, the fact that low quantum yield molecules spend less time in the first excited electronic state does not appear to make these molecules less prone to photobleaching than their fluorescent counterparts. Our data show that while this argument applies for one-photon excitation conditions, under the pulsed excitation that is necessary for EPR-SRS, also low quantum yield chromophores bleach significantly.

Despite these problems, our cellular imaging results show that EPR-SRS is a promising way to significantly increase the sensitivity of SRS miroscopy. We demonstrate that this is true for imaging of fixed and live cells using direct labeling as well as bioorthogonal labeling strategies with sensitivities similar to fluorescence imaging. Since EPR-SRS imaging is easily combined with confocal multiphoton fluorescence imaging, this opens exciting possibilities for multimodal imaging of biological samples.

## Supporting Information Available

Spontaneous Raman spectra of the quenchers, power dependency measurements of the background signal, time-resolved SRS data, fluorescence lifetime measurements, UV–vis spectra and photobleaching measurements, SRL spectrum of BBQ, SRS spectra of MB660R and RedDot2 (PDF)
