# Supplementary material for: Electronically Preresonant Stimulated Raman Scattering Microscopy of Weakly Fluorescing Chromophores
Source: J Phys Chem B. 2023 Jul 5;127(27):6029–37. doi: 10.1021/acs.jpcb.3c01407 (PMC10350953; doi:10.1021/acs.jpcb.3c01407)
Supplement: Supplementary file 1 — jp3c01407_si_001.pdf [file jp3c01407_si_001.pdf]

Electronically Pre-Resonant Stimulated Raman Scattering Microscopy of Weakly Fluorescing Chromophores

Andrea Pruccoli, Mustafa Kocademir, Martin J. Winterhalder, and Andreas Zumbusch*

Department of Chemistry, Universität Konstanz, Konstanz, Germany

E-mail: andreas.zumbusch@uni-konstanz.de

Phone: +49 (0)7531 882027

Contents

Spontaneous Raman spectroscopy **S3**

Power dependence of the SRL background observed for BH2 and BBQ **S5**

Fitting of time-resolved SRS data **S6**

Fluorescence lifetime measurements **S7**

UV-vis spectra and photobleaching measurements **S9**

SRL spectrum of BBQ **S11**

epr-SRS spectra of additional commercial dyes **S12**

Spontaneous Raman spectroscopy

Spontaneous Raman spectra of the chromophores were recorded using a commercial Raman microscope (MonoVista CRS, Spectroscopy & Imaging) equipped with three cw lasers (488 nm, 633 nm, and 785 nm). The solution of the chromophores in DMSO-d_6 was placed on a microscope slide and the Raman signal was detected in reflection after rejection of the Rayleigh scattered light with an appropriate bandpass filter. All spectra show a fluorescence background. In some cases this background is weak enough to allow clear identification of the Raman lines (BH2 and BBQ, Figure S1a,c), while in the other cases it dominates the Raman signal (BH3 and QSY21, Figure S1b,d).

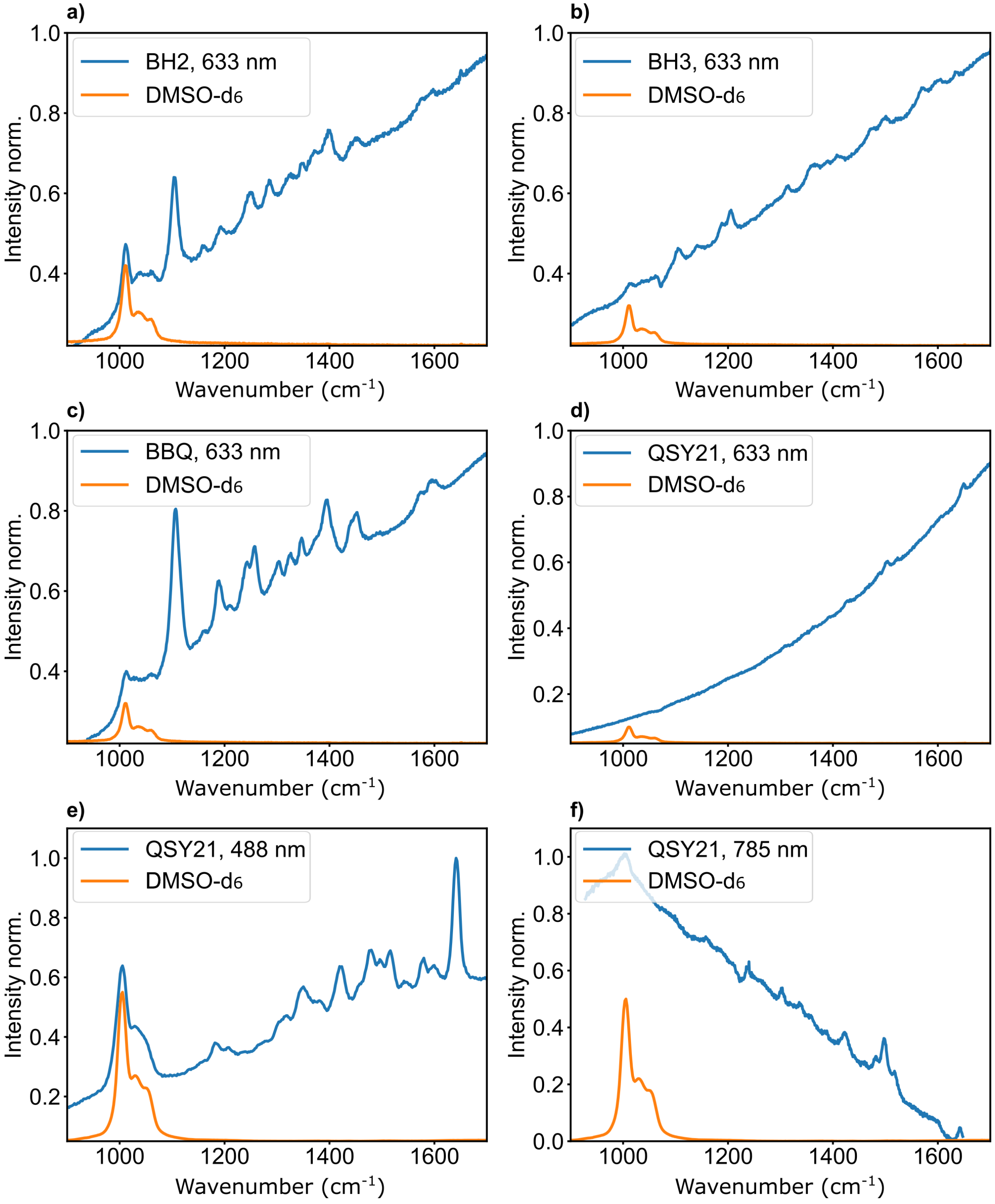

Figure S1: Resonance Raman spectra of the different quenchers investigated recorded with 633 nm excitation: a) Black Hole 2 (BH2), b) Black Hole 3 (BH3), c) Black Berry Quencher (BBQ), d) QSY21. e,f) Raman spectra of QSY21 recorded with 488 and 785 nm excitation, respectively. For the measurements, all compounds were dissolved in DMSO-d_6, displayed in orange (not in scale).

Power dependence of the SRL background observed for BH2 and BBQ

To better understand the origin of the background observed in the time-resolved SRS measurements, we carried out excitation power dependent measurements of 1 mM solutions of BH3 (Figure S2a,b) and BBQ (Figure S2c,d) in DMSO-d_6. In a first set of experiments, we recorded the sample fluorescence observed for irradiation with the Stokes beam at 840nm for BBQ and 860nm for BH3 (Figure S2a,c). The Stokes beam power was varied by a ND wheel and the fluorescence was recorded by an avalanche photodiode (τ-SPAD, PicoQuant). Power-dependent measurements of the SRL background were carried out by changing the power of the pump beam while a constant Stokes power was used. The pump loss signal was recorded by the SRS detector while the temporal delay between the two pulses was kept at -5 ps, such that no SRS or mixed two photon absorption could occur (Figure S2b,d).

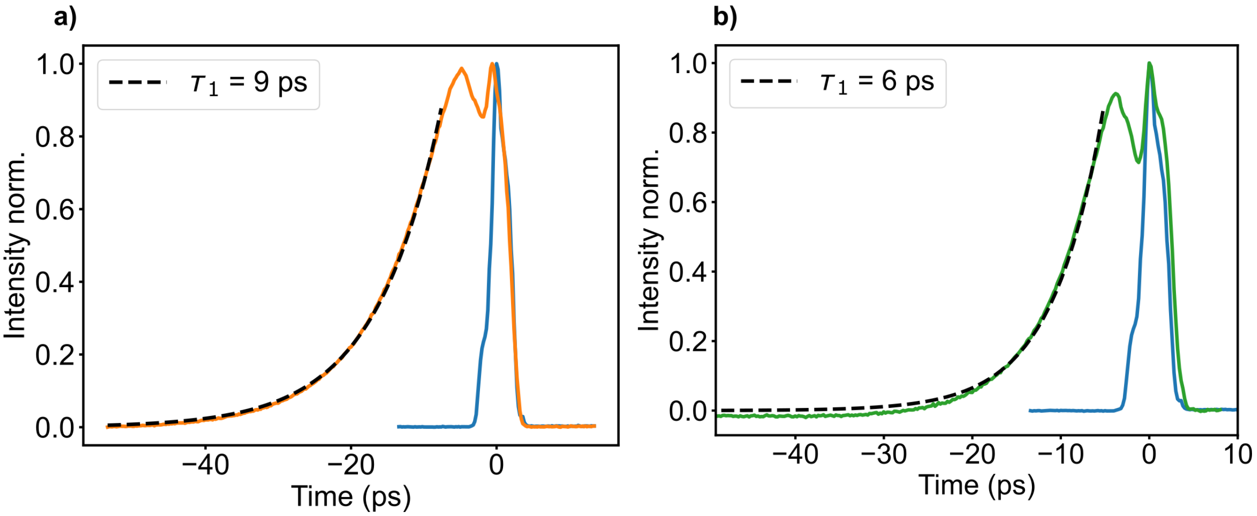

Figure S2: Dependence of the fluorescence intensity on the Stokes excitation power (a), c)) and dependence of the SRS background on pump excitation power (b), d)) for solutions of BH3 and BBQ, respectively.

Fitting of time-resolved SRS data

As discussed in the main text, time-resolved SRL data recorded for BH3 and BBQ exhibit a wavelength dependent background. For its analysis, we recorded the temporal correlation between the pump beam at 771 nm and the Stokes tuned to the strongest vibrational band of a 1 mM solution of the dye in DMSO-d_6. The rising signal at negative times (Stokes pulse before pump pulse) is tail-fitted with a simple exponential function yielding time constants of 6 and 9 ps, respectively. We attribute this signal to thermal cooling of the $|S_0\rangle$ state. For comparison, we also recorded time-resolved data for Rhodamine 800 that does not show a similar background.

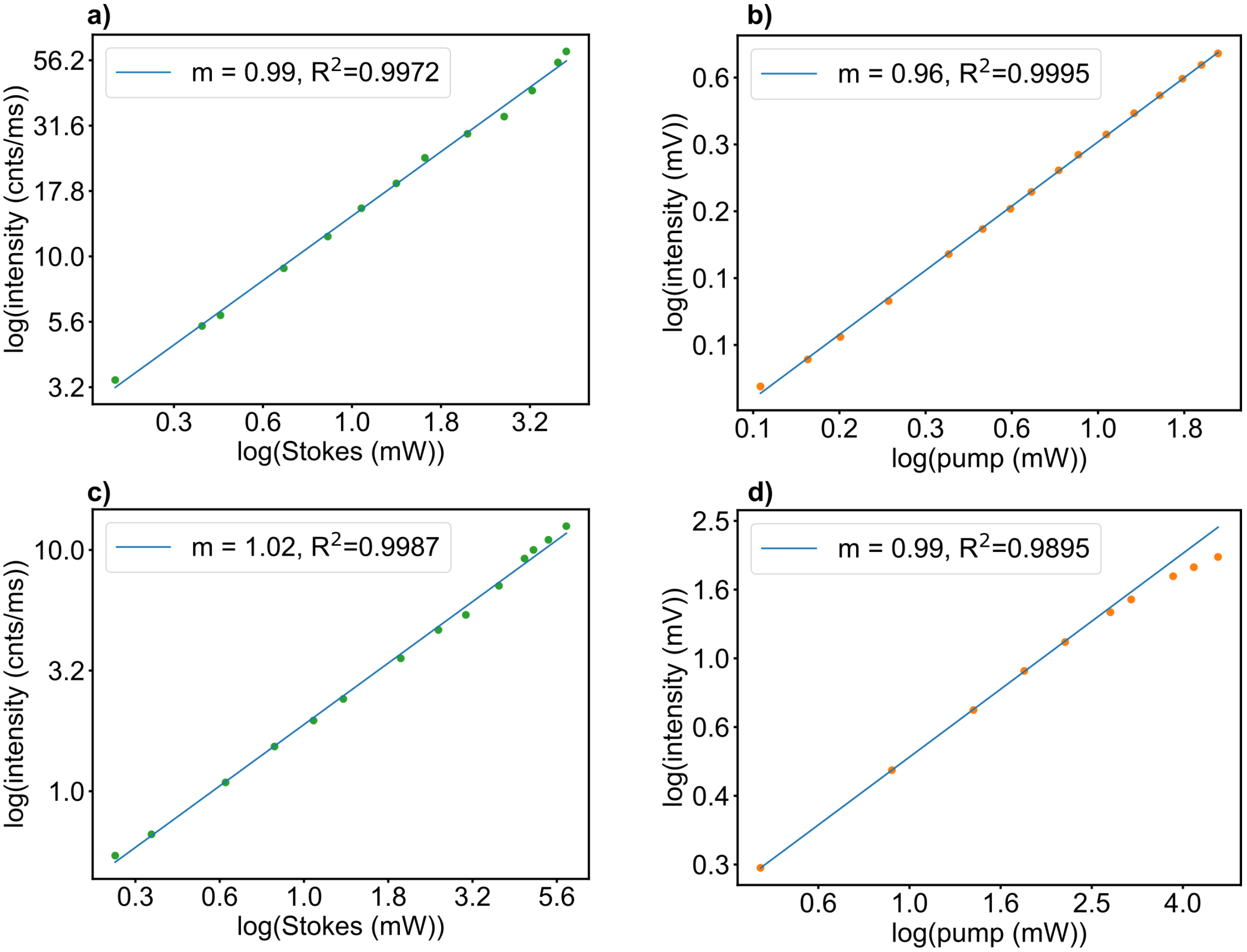

Figure S3: Time-resolved SRL data for a solution of Black Hole 3 (orange), Black Berry Quencher (green), and Rhodamine 800 (blue). The lifetime of the excited state is obtained by tail-fitting the curves with a simple exponential function.

Fluorescence lifetime measurements

The chromophores tested exhibit very weak fluorescence that allows fluorescence lifetime measurements for the confirmation of their short excited state lifetimes. The measurements were performed using a commercial TCSPC module (PicoHarp 300, PicoQuant). The reference signal was collected by a fast photodiode at 20MHz. The instrument response function (IRF) was measured with a sample of LUDOX solution using the scattered pump beam light at 771 nm. The weak fluorescence obtained by excitation with the pump beam was collected by an avalanche photodiode detector (τ-SPAD, PicoQuant). The power of the pump beam was adjusted to obtain 100 kcnts/s or less and the data collection was carried out until the decay curve would reach 10^4 counts. The lifetimes were then obtained by biexponential reconvolution fitting of the lifetime trace and the IRF.

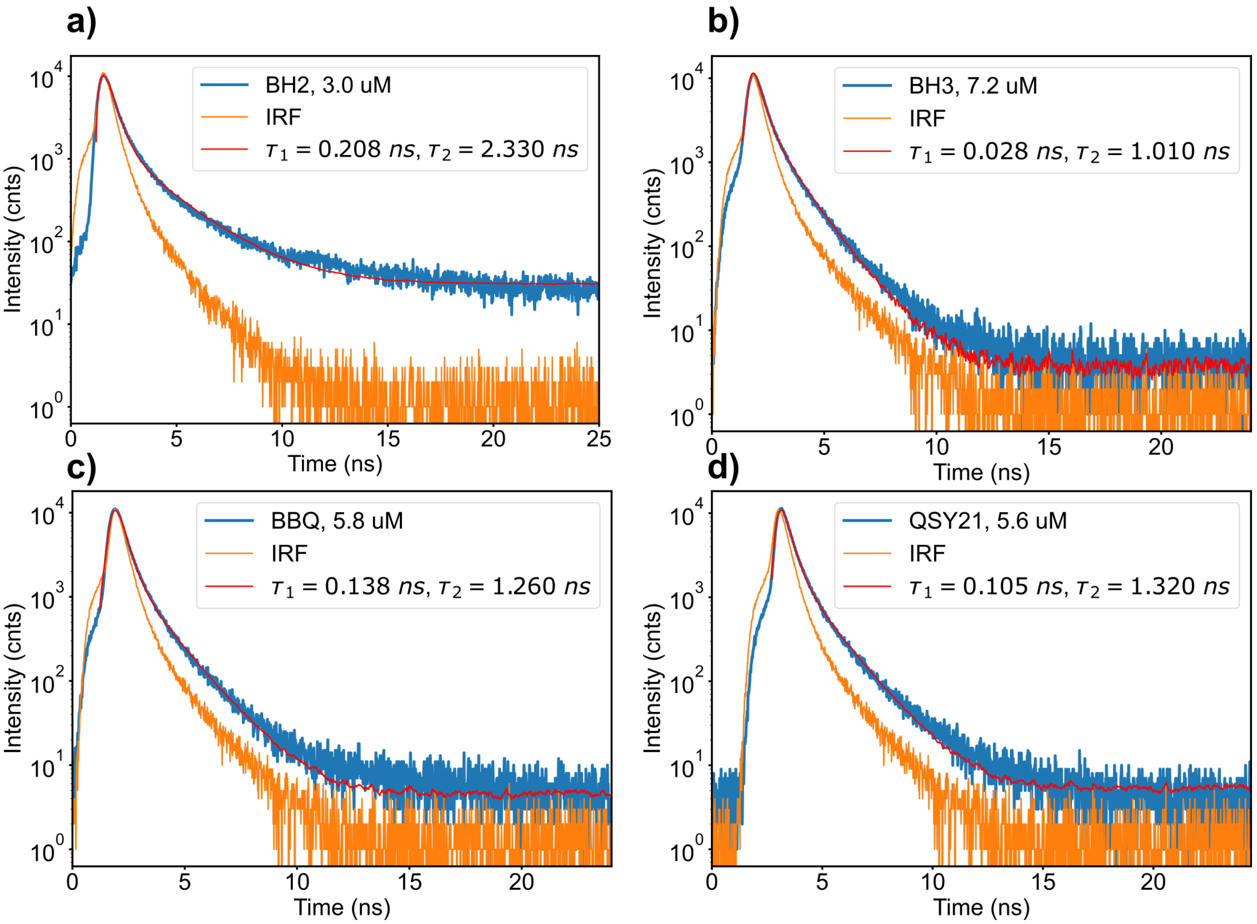

Figure S4: Fluorescence TCSPC measurements of a) BH2, b) BH3, c) BBQ, d) QSY21 (blue). IRF from LUDOX solutions (orange) and biexponential reconvolution fitting (red).

UV-vis spectra and photobleaching measurements

UV-vis absorption spectra of the dye solution in DMSO were recorded with a commercial UV-vis spectrometer (Cary50, Agilent Technologies) using 500 μL quartz cuvettes with 1 cm length. Photobleaching experiments were carried out by irradiating 500 μL solutions of the dyes in DMSO (5 μM) in quartz cuvettes with 7 mW of 633 nm light from a cw He-Ne laser. The absorption of the samples were measured at the beginning of the experiment and after every hour for a total of 6 hours. To confirm photobleaching a reference sample to which no irradiation was applied was measured in parallel.

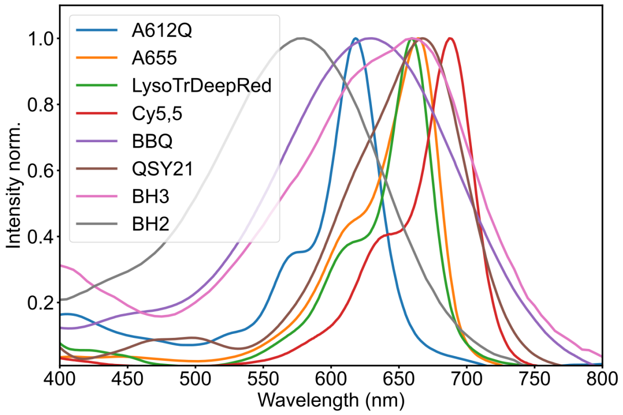

Figure S5: UV-vis spectra of Atto612Q (A612Q), Atto655 (A655), Lysotracker Deep Red studied (LysoTrDeepRed), Cyanine5.5 (Cy5,5), Black Berry Quencher (BBQ), QSY21, Black Hole 3 (BH3), Black Hole 2 (BH2) in DMSO.

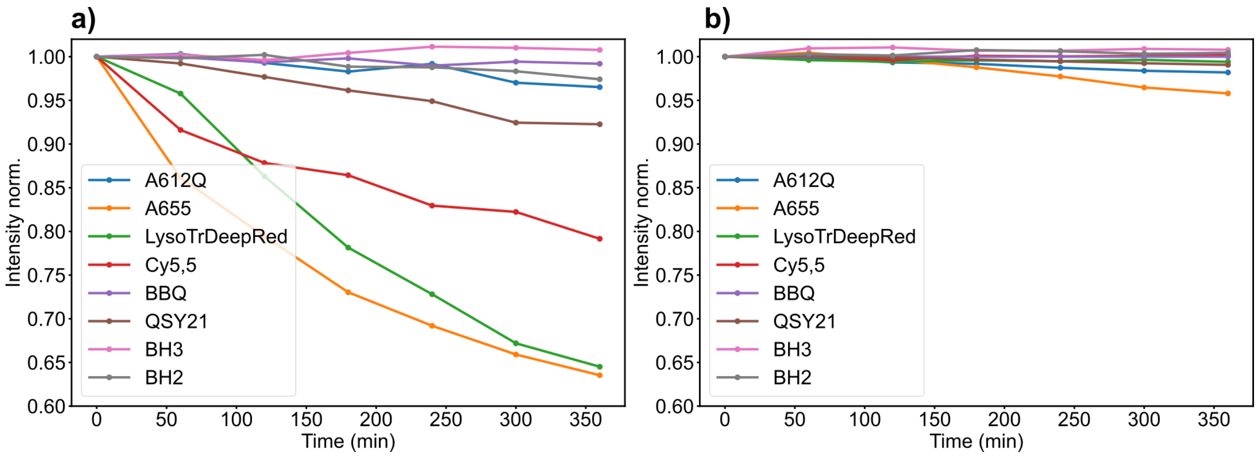

Figure S6: UV-vis absorption measurements while bleaching with a) a 633 nm cw laser and b) reference measurement of samples not exposed to irradiation.

SRL spectrum of BBQ

The SRL spectrum of BBQ was recorded with the homebuilt SRS microscope described in the main text (Figure S7a) using a pump beam at 771 nm and variable Stokes wavelengths. As a comparison, we also recorded an SRL spectrum using the commercial SRS microscope (TCS SP8, Leica Microsystem GmbH) with a variable pump wavelength and a Stokes wavelength fixed at 1030 nm (Figure S7b). In both cases a 1mM, BBQ solution in DMSO-d_6 with the homebuilt system and the commercial system. We can see that the transient background disappear moving from a pump at 771 nm to 925 nm at the expense of around 30% loss of signal intensity of the peak at 1100 cm^{-1} due to a partial loss of pre-resonance enhancement as we move further away from resonance.

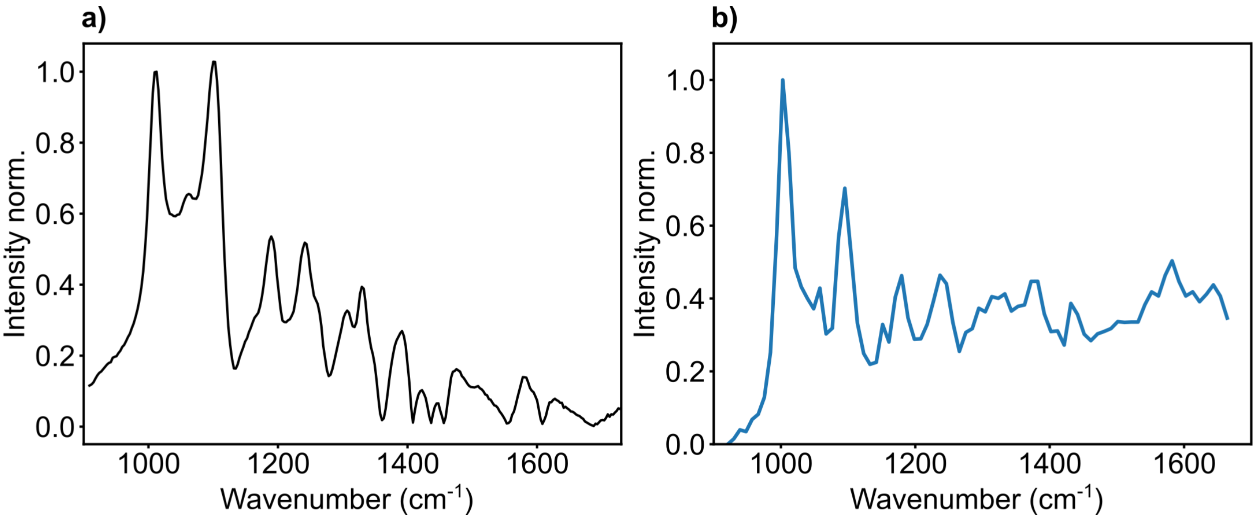

Figure S7: Stimulated Raman Scattering Spectrum of a 1 mM solution of Black Berry Quencher in DMSO-d_6 with homebuilt setup with fixed pump at 771 nm (a.) and commercial system with fixed Stokes at 1031 nm (b.). Both spectra are normalized with the solvent peak at 1000 cm^{-1}.

epr-SRS spectra of additional commercial dyes

The additional commercial dyes used for biological staining were first measured in the solution to obtain the SRS spectrum. MB660R (Click Chemistry Tools, US) with DBCO modification was prepared to a 1 mM solution in DMSO, fig.S8a. RedDot2 (Biotium, Inc., US) was purchased directly in solution with unknown concentration in DMSO.

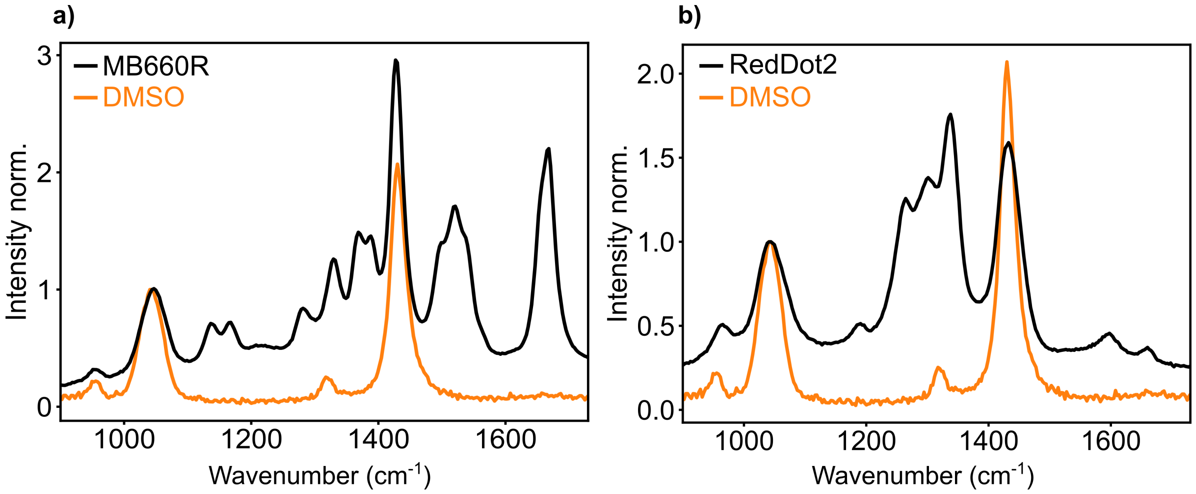

Figure S8: SRS spectrum of MB660R and RedDot2 solutions in DMSO. Normalized for the 1019 cm^{-1} peak of DMSO.
